# Global Burden of Female Breast Cancer: Age-Period-Cohort Analysis of Incidence Trends From 1990 to 2019 and Forecasts for 2035

**DOI:** 10.3389/fonc.2022.891824

**Published:** 2022-06-09

**Authors:** Yizhen Li, Jinxin Zheng, Yujiao Deng, Xinyue Deng, Weiyang Lou, Bajin Wei, Dong Xiang, Jingjing Hu, Yi Zheng, Peng Xu, Jia Yao, Zhen Zhai, Linghui Zhou, Si Yang, Ying Wu, Huafeng Kang, Zhijun Dai

**Affiliations:** ^1^ Department of Breast Surgery, The First Affiliated Hospital, College of Medicine, Zhejiang University, Hangzhou, China; ^2^ Department of Oncology, The Second Affiliated Hospital of Xi’an Jiaotong University, Xi’an, China; ^3^ Department of Nephrology, Ruijin Hospital, Institute of Nephrology, Shanghai Jiao Tong University School of Medicine, Shanghai, China; ^4^ Celilo Cancer Center, Oregon Health Science Center affiliated Mid-Columbia Medical Center, The Dalles, OR, United States; ^5^ Dana-Farber Cancer Institute, Harvard Medical School, Boston, MA, United States

**Keywords:** breast cancer, global burden of disease, age-period-cohort model, prediction, epidemiology

## Abstract

**Introduction:**

This study aimed to describe the latest epidemiology of female breast cancer globally, analyze the change pattern of the incidence rates and the disease’s association with age, period, and birth cohort, and subsequently present a forecast of breast cancer incidence.

**Methods:**

Data for analysis were obtained from Global Burden of Disease (GBD) Study 2019 and World Population Prospects 2019 revision by the United Nations (UN). We described the age-standardized incidence rates (ASIRs) from 1990 to 2019 and then calculated the relative risks of period and cohort using an age-period-cohort model, and predicted the trends of ASIRs to 2035.

**Results:**

In 2019, the global incidence of breast cancer in women increased to 1,977,212 (95% uncertainty interval = 1 807 615 to 2 145 215), with an ASIR of 45.86 (41.91 to 49.76) per 100 000 person-year. Among the six selected countries facing burdensome ASIRs, only the USA showed a downward trend from 1990 to 2019, whereas the others showed an increasing or stable trend. The overall net drift was similar in Japan (1.78%), India (1.66%), and Russia (1.27%), reflecting increasing morbidity from 1990 to 2019. The increase in morbidity was particularly striking in China (2.60%) and not significant in Germany (0.42%). The ASIRs were predicted to continue to increase globally, from 45.26 in 2010 to 47.36 in 2035. In most countries and regions, the age specific incidence rate is the highest in those aged over 70 years and will increase in all age groups until 2035. In high-income regions, the age specific incidence rates are expected to decline in women aged over 50 years.

**Conclusions:**

The global burden of female breast cancer is becoming more serious, especially in developing countries. Raising awareness of the risk factors and prevention strategies for female breast cancer is necessary to reduce future burden.

## Introduction

In 2020, female breast cancer surpassed lung cancer to become the leading cause of cancer incidence worldwide; an estimated 2.3 million new cases, accounting for 11.7% of all cancer cases, were reported (1). It is the fifth leading cause of cancer-related deaths worldwide, killing 685 000 patients. In women, breast cancer accounts for approximately a quarter of all cancer cases and a sixth of all cancer deaths, ranking first for incidence in 159 of 185 countries and for mortality in 110 countries (2). The incidence of breast cancer are higher and the death rates lower in developed countries than in developing countries (3). The high morbidity in more developed countries reflects the long-standing reproductive and hormonal risk factors (e.g., early menarche, late menopause, late first pregnancy, fewer births, hormone replacement therapy) and lifestyle factors (e.g., alcohol intake, excess body weight, lack of exercise) (4), as well as improved detection rates through mammographic screening (5).

To date, the vast majority of breast cancer-related studies have focused on pathogenesis and treatment regimens. Epidemiological studies could help shed light on the burden of breast cancer on human health from a macro perspective and provide bases for improving public health at the national level, allocating medical resources reasonably, and formulating health strategies. However, most of the relevant studies are limited to country- or region-specific analyses. In-depth analyses on the effects of age, period, and birth cohort have been lacking as well, as are comparisons of multiple countries. Moreover, no studies have focused on the prediction of the incidence of female breast cancer at the global and regional levels. The Global Burden of Disease Study (GBD) database and the United Nations (UN) population data provide researchers with the possibility of deep analysis and forecasting (6).

In the present study, we obtained data from the GBD 2019, combined with UN standard and predicted population data, to describe the latest epidemiology of female breast cancer worldwide, analyze the change pattern of the incidence rates as well as the disease’s association with age, period, and birth cohort in six selected countries with large population, and present forecasts for global, regional, and selected countries’ incidence up to 2035.

## Methods and Materials

### Data Source

We obtained data on the incidence of and deaths from breast cancer from the Global Health Data Exchange GBD Results Tool (http://ghdx.healthdata.org/gbd-results-tool), which is a catalog of global health and demographic data created by GBD collaborators. This tool provides a comparative assessment of health loss from 369 diseases and 87 risk factors across 204 countries and territories within 21 GBD regions (2). The age-standardized incidence rate (ASIR) and age-standardized death rate (ASDR) of breast cancer were also obtained by sex (female only), location, age, year (from 1990 to 2019), and social-demographic index (SDI), reported with their 95% uncertainty intervals (UIs). According to the World Bank (WB) income levels, the world is divided into four regions: high, upper middle, lower middle, and low income regions. Current and projected population data by country, year, sex (female only), and age were obtained from the World Population Prospects 2019 revision (https://population.un.org/wpp/), which is an official UN population estimates and projections prepared by the Population Division of Economic and Social Affairs Department of the UN Secretariat (7).

### Statistical Analysis

Of the ten countries with the highest incidence and mortality of female breast cancer, we selected six countries with the highest burden of breast cancer, namely, China, Germany, India, Japan, the Russian Federation, and the USA. We conducted incidence prediction for 13 countries, which were selected by taking the union set of the top 10 countries for incidence, deaths, prevalence, and disability-adjusted life years of female breast cancer (see **Supplementary Table 1**).

We applied age-period cohort (APC) modeling to assess the associations among age, period, birth cohort, and breast cancer morbidity; an individual’s birth cohort could be calculated by the time period of diagnosis and their age (birth cohort = period - age) (8). Period effects reflect changes over time, which affect all age groups concurrently, presumably arising from changes in social, economic, cultural, or physical environments. Cohort effects are associated with changes in individual groups with the same birth year (9). Period and cohort relative risks (RRs) represent the ratios of age-specific rates in each period and each cohort relative to a reference group, respectively. The age curve indicates the expected age-specific rate in a reference cohort adjusted for period effects. Net and local drifts are two important parameters in APC models: the former refers to the overall log-linear trend by period and birth cohort, and indicates the overall annual percentage change of the expected age-adjusted rates over time; the latter represents the log-linear trend by period and birth cohort for each age group and indicates the annual percentage change in the expected age-specific rates over time.

For APC analysis, we arranged the morbidity and population data into successive five-year periods: from 1990–1994 (median 1992) to 2015–2019 (median 2017), with 2000–2004 as the reference period, and successive age groups with five-year age intervals from 20–24 years to 75–79 years. Thus, we created 17 consecutive birth cohorts, including those born in 1908–1912 (median 1910) to 1988–1992 (median 1990), with the birth cohort 1958–1962 (median 1960) as the reference group. We obtained the estimated parameters from the APC web tool provided by the National Cancer Institute (8).

To predict the number of new cases and incidence rates of female breast cancer from 2020 to 2035 by country, sex (female only), and age, we applied a log-linear APC model with limited linear trend prediction to fit the recent trends (10). The prediction was conducted in R 4.0.3 through the NORDPRED package, which performs well in predicting future trends in cancer incidence (11). Incidence rates were calculated by the five-year period of diagnosis from 1908–1912 (median 1910) to 1988–1992 (median 1990) and five-year age groups for all ages (from 20–24 to 75–79 years). We calculated the ASIRs using the world standard population, based on the latest United Nation population assessment in 1998 (12). Thus, we predicted the number of new cases for the year 2035 by taking a weighted average of the predicted incidence rates centering on 2035 and then applying the rates to the UN national population forecasts for each country for that year. All data calculated according to the APC model were reported with their 95% confidence intervals (CIs). We used the Wald chi-squared test to test the significance of the estimated parameters and functions. All statistical tests were two-tailed.

### Data Disclosure Statement

This study is based on the GBD 2019 database, which does not contain identifiable personal information; therefore, a waiver of informed consent was reviewed and approved by the Institutional Review Board of the University of Washington.

## Results

### Overall Description of Female Breast Cancer

**Figure 1:**
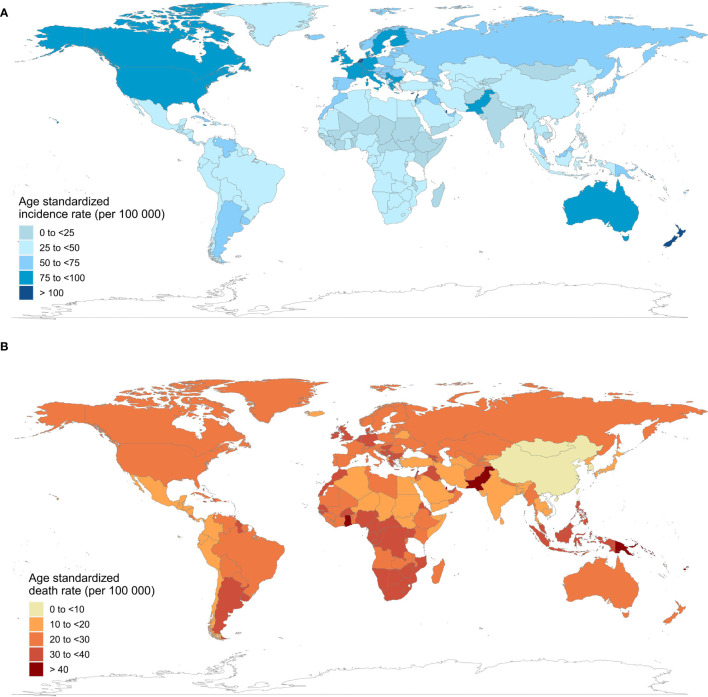
Age-standardized incidence and death rates of female breast cancer in 204 countries and territories, 2019. **(A)** age-standardized incidence rates; **(B)** age-standardized death rates.

**Table 1** shows the current incidence and death cases of female breast cancer, and their ASIRs, along with the percentage changes from 2010 to 2019. In 2019, the global incidence of breast cancer in women increased to 1 977 212 (95% UI = 1 807 615 to 2 145 215), with an ASIR of 45.86 (95% UI = 41.91 to 49.76) per 100 000 person-year. The number of breast cancer deaths increased to 688 562 (95% UI = 635 323 to 739 571) in 2019, whereas the ASDR decreased by 1.86% (95% UI = -7.89% to 4.15%) compared with 2010. Regionally, within the five SDI quintiles, the ASIR of female breast cancer decreased from the high SDI quintile (79.22 per 100 000 person-year) to the low SDI quintile (25.67), whereas the ASDR was the highest in the low SDI quintile (18.34) and lowest in the middle SDI quintile (13.66). Among the 21 regions in the GBD, high-income North America (93.75), Western Europe (85.85), and Australasia (84.69) had the highest ASIRs, whereas Oceania (42.80), Southern Latin America (24.04), and Western Sub-Saharan Africa (23.25) had the highest ASDRs. At the national level, China (incidence = 368 375, deaths = 93 499), the USA (incidence = 251 531, deaths = 54 402), and India (incidence = 144 086, deaths = 82 099) also had the highest number of new cases and deaths from breast cancer in women. Monaco (149.60), Solomon Islands (126.48), and Lebanon (122.51) had the highest ASIRs among the 204 countries and territories worldwide, and Solomon Islands (75.04), Pakistan (51.94), and Papua New Guinea (43.47) had the highest ASDRs in 2019 (**Figure 1**).

**Table 1:** The incidence and death cases and their age-standardized rates of female breast cancer in 2010 and 2019, and the percentage changes from 2010 to 2019.

Location	2010				2019				Percentage change 2010 to 2019	
	Incidence No. × 10^3^(95% UI)	ASIR per 100,000(95% UI)	Deaths No. × 10^3^(95% UI)	ASDR per 100,000(95% UI)	Incidence No. × 10^3^(95% UI)	ASIR per 100,000(95% UI)	Deaths No. × 10^3^(95% UI)	ASDR per 100,000(95% UI)	ASIR(95% UI)	ASDR(95% UI)
**Global**	1563.6 (1505.3 to 1615.5)	45.07 (43.34 to 46.56)	554.7 (526.3 to 576.6)	16.18 (15.32 to 16.83)	1997.2 (1807.6 to 2145.2)	45.86 (41.91 to 49.76)	688.6 (635.3 to 739.6)	15.88 (14.66 to 17.07)	1.75 (-5.94 to 10.08)	-1.86 (-7.89 to 4.15)
**SDI**										
**High**	617.4 (588.7 to 635.8)	83.37 (80.27 to 85.52)	150.9 (138.7 to 157.2)	18.08 (17.00 to 18.67)	673.1 (601.3 to 747.7)	79.22 (70.83 to 87.70)	166.0 (150.3 to 175.2)	16.71 (15.56 to 17.45)	-4.98 (-14.80 to 4.93)	-7.57 (-10.30 to -4.65)
**High middle**	419.3 (402.0 to 435.5)	47.89 (45.93 to 49.75)	144.6 (137.2 to 150.4)	16.28 (15.45 to 16.94)	510.3 (458.4 to 568.0)	48.93 (43.84 to 54.49)	163.5 (150.5 to 177.2)	14.93 (13.75 to 16.19)	2.18 (-7.79 to 13.80)	-8.29 (-14.98 to -0.63)
**Middle**	331.8 (313.6 to 351.2)	30.76 (29.08 to 32.55)	138.2 (130.7 to 146.3)	13.58 (12.83 to 14.39)	485.8 (430.2 to 545.2)	35.52 (31.47 to 39.81)	181.1 (162.7 to 201.7)	13.66 (12.30 to 15.18)	15.45 (2.56 to 29.69)	0.55 (-9.36 to 11.03)
**Low middle**	144.3 (133.7 to 155.8)	24.28 (22.49 to 26.17)	85.7 (78.5 to 93.3)	15.21 (13.95 to 16.53)	227.2 (199.1 to 256.0)	29.47 (25.91 to 33.20)	124.9 (108.0 to 142.6)	16.86 (14.59 to 19.24)	21.41 (7.75 to 36.28)	10.81 (-3.52 to 25.23)
**Low**	49.8 (44.1 to 56.0)	21.75 (19.16 to 24.39)	35.0 (30.9 to 39.2)	16.46 (14.56 to 18.41)	79.4 (69.2 to 90.9)	25.67 (22.54 to 29.10)	52.5 (45.7 to 60.0)	18.34 (15.98 to 20.84)	18.03 (4.07 to 32.90)	11.43 (-1.59 to 25.19)
**GBD region**										
**Andean Latin America**	6.2 (5.4 to 7.0)	27.07 (23.70 to 30.61)	2.9 (2.6 to 3.3)	13.22 (11.65 to 14.76)	9.0 (7.3 to 11.0)	29.63 (24.05 to 36.45)	3.8 (3.1 to 4.6)	12.67 (10.44 to 15.51)	9.44 (-12.72 to 36.74)	-4.16 (-21.90 to 17.32)
**Australasia**	16.8 (15.6 to 17.8)	89.87 (84.21 to 95.08)	3.8 (3.5 to 4.0)	18.82 (17.45 to 19.69)	19.2 (15.5 to 23.7)	84.69 (68.25 to 104.99)	4.4 (4.0 to 4.8)	17.47 (16.11 to 18.69)	-5.76 (-24.06 to 16.93)	-7.18 (-12.05 to -1.62)
**Caribbean**	12.1 (11.3 to 13.0)	53.53 (49.95 to 57.43)	4,7 (4.3 to 5.2)	20.76 (19.07 to 22.74)	14.9 (12.6 to 17.6)	55.37 (46.63 to 65.11)	5.7 (4.8 to 6.7)	20.84 (17.62 to 24.40)	3.44 (-11.28 to 20.44)	0.37 (-12.13 to 14.48)
**Central Asia**	13.9 (13.4 to 14.5)	37.50 (35.99 to 39.04)	6.6 (6.3 to 6.8)	18.72 (18.03 to 19.42)	17.7 (15.8 to 19.9)	38.36 (34.23 to 42.80)	7.5 (6.7 to 8.4)	17.29 (15.53 to 19.16)	2.31 (-8.57 to 14.07)	-7.61 (-16.24 to 2.34)
**Central Europe**	56.4 (54.5 to 58.0)	59.76 (57.93 to 61.44)	22.0 (20.9 to 22.5)	21.22 (20.31 to 21.74)	60.8 (52.6 to 69.9)	60.22 (52.04 to 69.57)	23.0 (20.1 to 26.2)	19.87 (17.25 to 22.71)	0.76 (-12.41 to 16.82)	-6.35 (-18.19 to 7.00)
**Central Latin America**	34.8 (33.6 to 35.8)	34.54 (33.23 to 35.57)	12.3 (11.8 to 12.6)	12.65 (12.05 to 13.03)	50.6 (42.5 to 60.0)	38.45 (32.30 to 45.64)	16.7 (14.3 to 19.6)	12.87 (11.05 to 15.09)	11.33 (-6.04 to 32.09)	1.77 (-12.62 to 18.65)
**Central Sub-Saharan Africa**	5.8 (4.6 to 7.1)	23.47 (18.91 to 28.61)	4.3 (3.5 to 5.3)	19.06 (15.39 to 23.08)	9.7 (7.0 to 12.8)	28.98 (20.86 to 38.55)	6.8 (5.0 to 9.0)	22.42 (16.16 to 29.76)	23.45 (-1.66 to 53.70)	17.65 (-5.86 to 44.68)
**East Asia**	257.8 (228.6 to 289.0)	29.62 (26.35 to 33.19)	75.1 (67.3 to 84.1)	9.06 (8.15 to 10.11)	382.3 (303.3 to 477.2)	35.69 (28.32 to 44.54)	98.2 (79.2 to 120.1)	9.12 (7.36 to 11.13)	20.47 (-5.87 to 52.35)	0.63 (-21.58 to 25.94)
**Eastern Europe**	87.4 (85.6 to 88.8)	50.59 (49.67 to 51.39)	35.8 (34.8 to 36.5)	19.24 (18.72 to 19.56)	94.0 (80.4 to 110.3)	51.89 (44.14 to 61.31)	35.0 (30.3 to 40.4)	17.47 (15.05 to 20.36)	2.57 (-12.79 to 21.13)	-9.19 (-21.79 to 5.49)
**Eastern Sub-Saharan Africa**	14.6 (12.6 to 16.8)	20.26 (17.57 to 23.17)	10.7 (9.3 to 12.3)	16.18 (14.07 to 18.39)	23.9 (20.2 to 27.8)	24.04 (20.78 to 27.49)	16.4 (14.0 to 18.9)	18.15 (15.65 to 20.60)	18.69 (4.58 to 33.87)	12.20 (0.40 to 26.03)
**High-income Asia Pacific**	87.8 (81.3 to 92.5)	57.22 (54.00 to 59.79)	18.2 (16.3 to 19.3)	10.45 (9.69 to 10.88)	97.2 (81.0 to 115.1)	56.30 (47.14 to 67.18)	20.5 (17.8 to 22.3)	9.78 (8.91 to 10.41)	-1.61 (-16.46 to 16.94)	-6.45 (-10.26 to -2.24)
**High-income North America**	251.4 (240.2 to 260.3)	98.54 (94.99 to 101.59)	54.0 (50.0 to 56.4)	19.35 (18.26 to 20.07)	280.0 (233.4 to 335.0)	93.75 (78.03 to 112.64)	60.9 (56.3 to 64.2)	18.36 (17.28 to 19.19)	-4.86 (-20.73 to 14.95)	-5.13 (-8.45 to -1.91)
**North Africa and Middle East**	60.1 (55.5 to 65.2)	32.04 (29.68 to 34.56)	26.2 (24.0 to 28.5)	15.15 (13.92 to 16.39)	94.7 (82.3 to 108.9)	37.48 (32.68 to 42.94)	35.4 (30.7 to 40.6)	15.22 (13.31 to 17.35)	16.98 (6.55 to 28.40)	0.48 (-8.39 to 10.06)
**Oceania**	2.0 (1.6 to 2.5)	57.72 (46.33 to 71.95)	1.2 (1.0 to 1.6)	39.09 (31.34 to 48.73)	3.0 (2.3 to 3.8)	65.58 (50.44 to 83.58)	1.8 (1.4 to 2.3)	42.80 (33.19 to 54.23)	13.61 (-4.26 to 36.02)	9.47 (-7.79 to 30.49)
**South Asia**	131.8 (119.2 to 145.4)	22.44 (20.23 to 24.72)	82.9 (74.4 to 91.6)	15.00 (13.37 to 16.65)	215.8 (178.1 to 256.9)	27.72 (22.91 to 33.00)	125.3 (103.1 to 149.4)	16.83 (13.91 to 20.00)	23.52 (1.46 to 49.92)	12.18 (-8.48 to 35.71)
**Southeast Asia**	101.2 (91.1 to 110.6)	35.10 (31.77 to 38.30)	52.7 (47.7 to 58.0)	19.39 (17.64 to 21.29)	138.5 (118.9 to 161.2)	38.52 (33.11 to 44.64)	66.5 (57.1 to 76.4)	19.23 (16.62 to 22.01)	9.74 (-5.19 to 28.44)	-0.84 (-14.69 to 14.41)
**Southern Latin America**	20.4 (19.4 to 21.3)	55.56 (53.00 to 57.93)	10.0 (9.4 to 10.4)	25.86 (24.57 to 26.78)	24.6 (19.2 to 31.1)	56.51 (43.78 to 71.94)	11.1 (10.3 to 11.9)	24.04 (22.41 to 25.61)	1.70 (-21.74 to 29.21)	-7.02 (-12.57 to -1.37)
**Southern Sub-Saharan Africa**	9.5 (8.5 to 10.6)	34.67 (31.20 to 38.58)	6.3 (5.7 to 6.9)	24.15 (21.97 to 26.63)	11.5 (10.3 to 13.0)	33.89 (30.14 to 38.02)	7.1 (6.3 to 7.9)	22.06 (19.72 to 24.54)	-2.24 (-13.22 to 10.54)	-8.67 (-18.14 to 1.63)
**Tropical Latin America**	42.8 (41.2 to 44.3)	41.19 (39.54 to 42.62)	17.3 (16.4 to 17.9)	17.10 (16.16 to 17.70)	53.2 (49.8 to 56.5)	39.75 (37.24 to 42.24)	20.3 (18.9 to 21.5)	15.19 (14.15 to 16.11)	-3.52 (-9.53 to 2.71)	-11.17 (-16.09 to -5.73)
**Western Europe**	327.4 (310.3 to 338.8)	91.79 (88.02 to 94.62)	92.0 (84.2 to 96.0)	21.66 (20.30 to 22.42)	338.6 (292.3 to 387.0)	85.85 (74.12 to 98.85)	97.5 (87.4 to 103.3)	19.79 (18.32 to 20.77)	-6.47 (-19.31 to 7.12)	-8.63 (-11.47 to -5.17)
**Western Sub-Saharan Africa**	23.4 (18.5 to 29.3)	28.33 (22.75 to 34.77)	15.7 (12.4 to 19.1)	20.71 (16.75 to 24.75)	38.0 (29.5 to 46.9)	32.91 (25.93 to 40.11)	24.5 (19.2 to 30.8)	23.25 (18.65 to 28.62)	16.16 (-10.59 to 46.38)	12.22 (-11.22 to 41.83)
**WB Income Level**										
**High**	739.2 (703.8 to 761.8)	82.61 (79.48 to 84.78)	188.2 (172.7 to 196.1)	18.53 (17.40 to 19.14)	800.2 (712.1 to 884.1)	78.70 (70.44 to 87.05)	206.0 (187.0 to 217.4)	17.16 (16.00 to 17.95)	-4.73 (-14.54 to 5.02)	-7.36 (-9.96 to -4.40)
**Low**	31.5 (27.5 to 36.0)	21.94 (19.30 to 24.88)	22.0 (19.4 to 25.0)	16.44 (14.60 to 18.51)	48.4 (40.1 to 58.5)	25.31 (21.11 to 30.38)	32.2 (27.0 to 38.6)	18.17 (15.37 to 21.58)	15.35 (2.16 to 30.82)	10.47 (-2.04 to 24.23)
**Upper middle**	505.2 (475.6 to 536.7)	35.09 (33.04 to 37.23)	177.0 (167.3 to 186.1)	12.74 (12.01 to 13.39)	695.7 (607.6 to 798.5)	39.16 (34.19 to 44.98)	215.2 (193.5 to 240.2)	12.08 (10.86 to 13.47)	11.60 (-2.59 to 28.37)	-5.11 (-15.67 to 6.46)
**Lower middle**	286.6 (267.5 to 306.3)	27.43 (25.61 to 29.29)	167.1 (155.6 to 179.4)	17.00 (15.82 to 18.30)	431.7 (383.1 to 481.1)	31.71 (28.16 to 35.32)	234.6 (206.2 to 263.5)	18.13 (15.90 to 20.30)	15.61 (2.55 to 28.81)	6.61 (-6.35 to 19.70)

GBD, global burden of disease; SDI, social-demographic index; UI, Uncertainty Interval; WB, world bank.

### Age-Period-Cohort Analysis for Six Selected Countries

**Figure 2:**
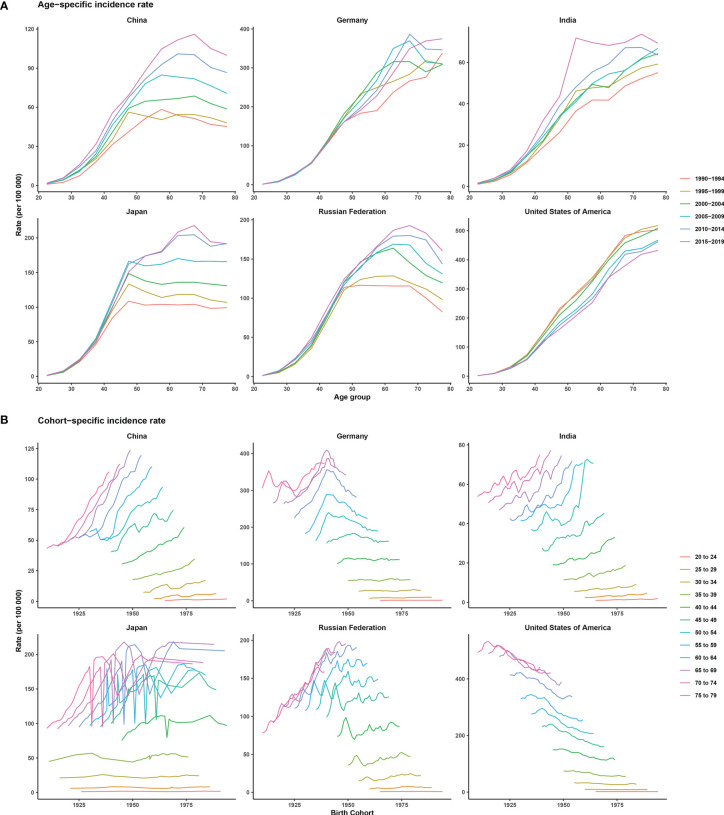
Age-specific incidence rates by period and cohort-specific incidence rates by age group of female breast cancer across six most burdensome countries from 1990 to 2019. Note: The six most burdensome countries include China, Germany, India, Japan, Russian Federation, and the United States of America. **(A)** age-specific incidence rate, survey years were arranged into six consecutive 5-year periods from 1990-1994 (median 1992) to 2015-2019 (median 2017), longitudinal age curves were estimated by age-period-cohort model and indicated the expected age-specific rates of incidence. **(B)** cohort-specific incidence rate, people aged 20 to 79 were divided into twelve consecutive 5-year age groups from 20-24 (median 22) to 75-79 (median 77), thus we have 17 consecutive birth cohorts, including those born from 1908-1912 (median 1910) to 1988-1992 (median 1990).

**Supplementary Figure 1** shows the trends in female breast cancer incidence rates across six selected countries. Generally, only the ASIR of the USA showed a downward trend from 1990 to 2019. Meanwhile, India and China were two countries where the ASIRs increased every year. ASIRs had remained stable or declined slightly in the Russian Federation, Japan, and Germany over the past decade. The peak incidence rate for China, Japan, and the Russian Federation occurred in people aged between 60 and 70 years across different historical periods, with the exception of two periods, 1990–1994 and 1995–1999. For Germany, India, and the USA, morbidity and age showed a roughly positive correlation in each historical period, but in the same age group in the USA, morbidity was higher in earlier periods. China, India, and the Russian Federation showed an increasing trend of breast cancer morbidity across birth cohorts, whereas Germany and Japan first showed a rise and then a decline across all age groups. The USA showed a downward trend across birth cohorts, indicating a relatively lower risk of morbidity in more recent birth cohorts (**Figure 2**).

**Figure 3:**
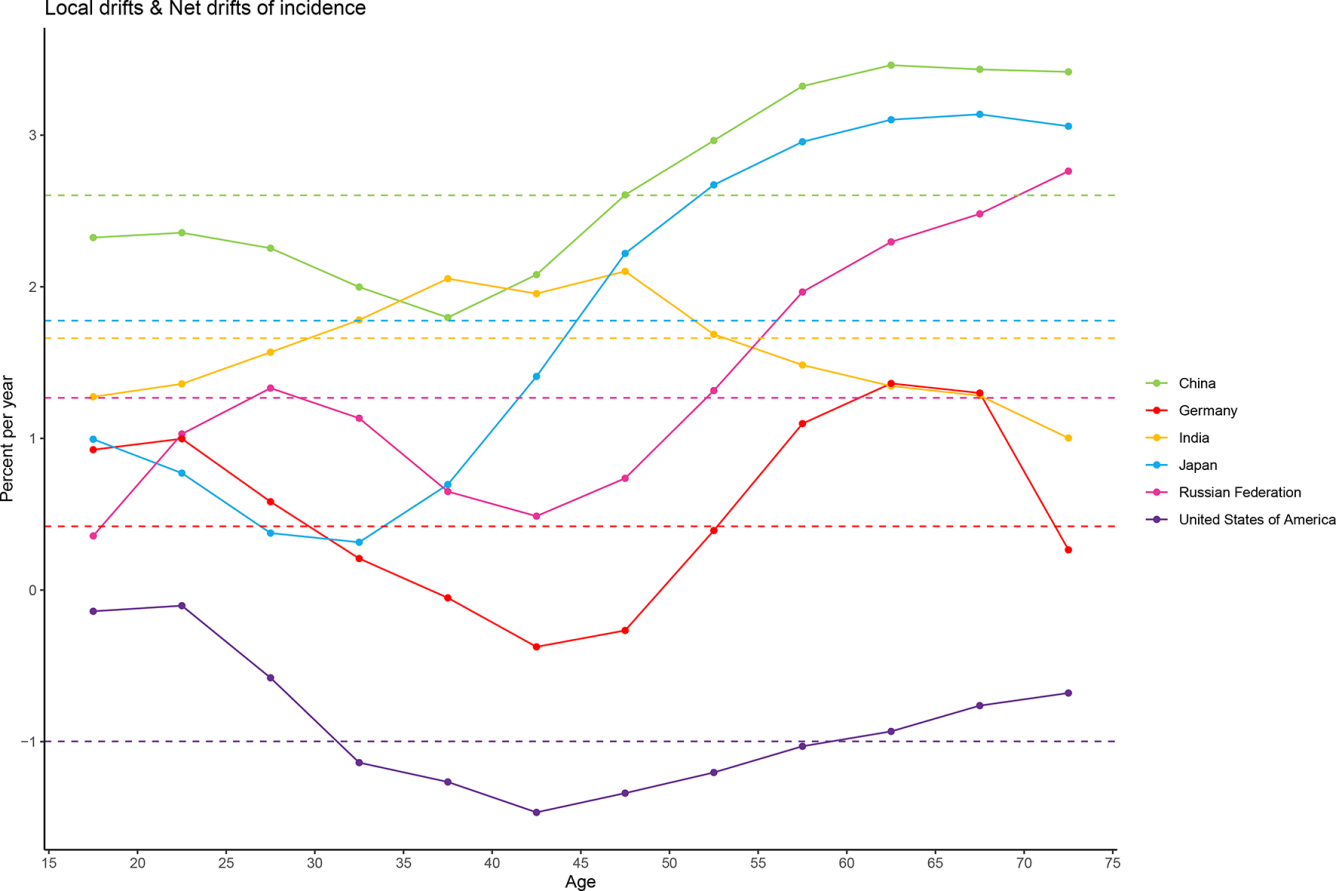
Local drift and net drift values for female breast cancer compared in the six most burdensome countries from 1990 to 2019. Note: Net drift indicates the overall annual percentage change over time, and local drift indicates the annual percentage change of each age group. The horizontal and dashed lines with different colors represent the levels of net drift in different countries, and the broken and solid lines represent the levels of local drift in different countries.

The overall net drift was similar in Japan (1.78%, 95% CI: 1.30% to 2.26%), India (1.66%, 95% CI: 0.95% to 2.37%), and the Russian Federation (1.27%, 95% CI: 0.74% to 1.80%), reflecting an increase in female breast cancer morbidity from 1990 to 2019. Morbidity increase was the most striking in China (2.60%, 95% CI: 2.00% to 3.21%) and not very significant in Germany (0.42%, 95% CI: -0.02% to 0.86%). Net drift in only the USA (-1.00%, 95% CI: -1.40% to -0.59) showed a significant decrease in morbidity. For local drift, values were predominantly above 0 for most age groups, except for all age groups in the USA (-1.47% to -0.10%) and women aged 35 to 50 years (-0.37% to -0.05%) in Germany (**Figure 3**).

**Figure 4:**
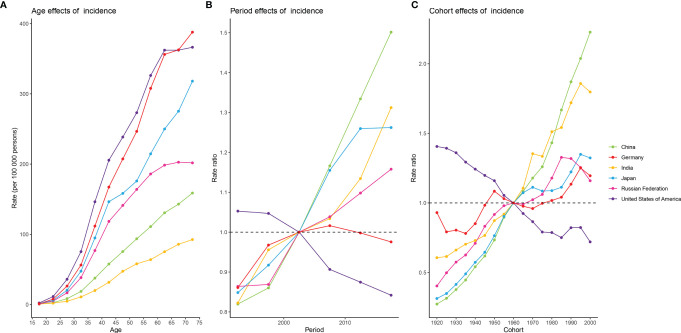
Estimates of age, period, and cohort effects on the incidence of female breast cancer in the six burdensome countries from 1990 to 2019. **(A)** age effects; **(B)** period effects, with period 2000-2004 (median 2002) as reference period; **(C)** cohort effects, with cohort born in 1958-1962 (median 1960) as reference birth cohort.

**Figure 4** shows estimates of APC effects on female breast cancer morbidity. Age effects across the six countries showed an expected build-up curve. The steepest increases with age were in the USA and Germany, whereas rates in Japan increased rapidly in the earlier years of life. Period and cohort effects tended to show similar patterns for the six countries. For China and India, period effects had been increasing over time, suggesting no improvements for the entire population across the study period. Improvements were observed only in the USA, where more favorable period trends occurred over the past decades. The most significant increase in RRs across birth cohorts was seen in China, with progressive increase in morbidity in those born after 1920. Cohort effects showed different directions for individual countries. The only striking improvements across birth cohort were in the USA, with significant reduction in morbidity in women born after 1920 onwards. For China and India, progressive increases in morbidity were observed in women born after 1920, whereas such a change was not evident in Germany. For Japan, and the Russian Federation, the aggravation in morbidity in older cohorts was less striking and appeared to have stalled for cohorts born after 1960. In addition, there was some indication of more favorable trends among women born after 1995 in the other five countries except China.

### Overall Prediction

**Figure 5:**
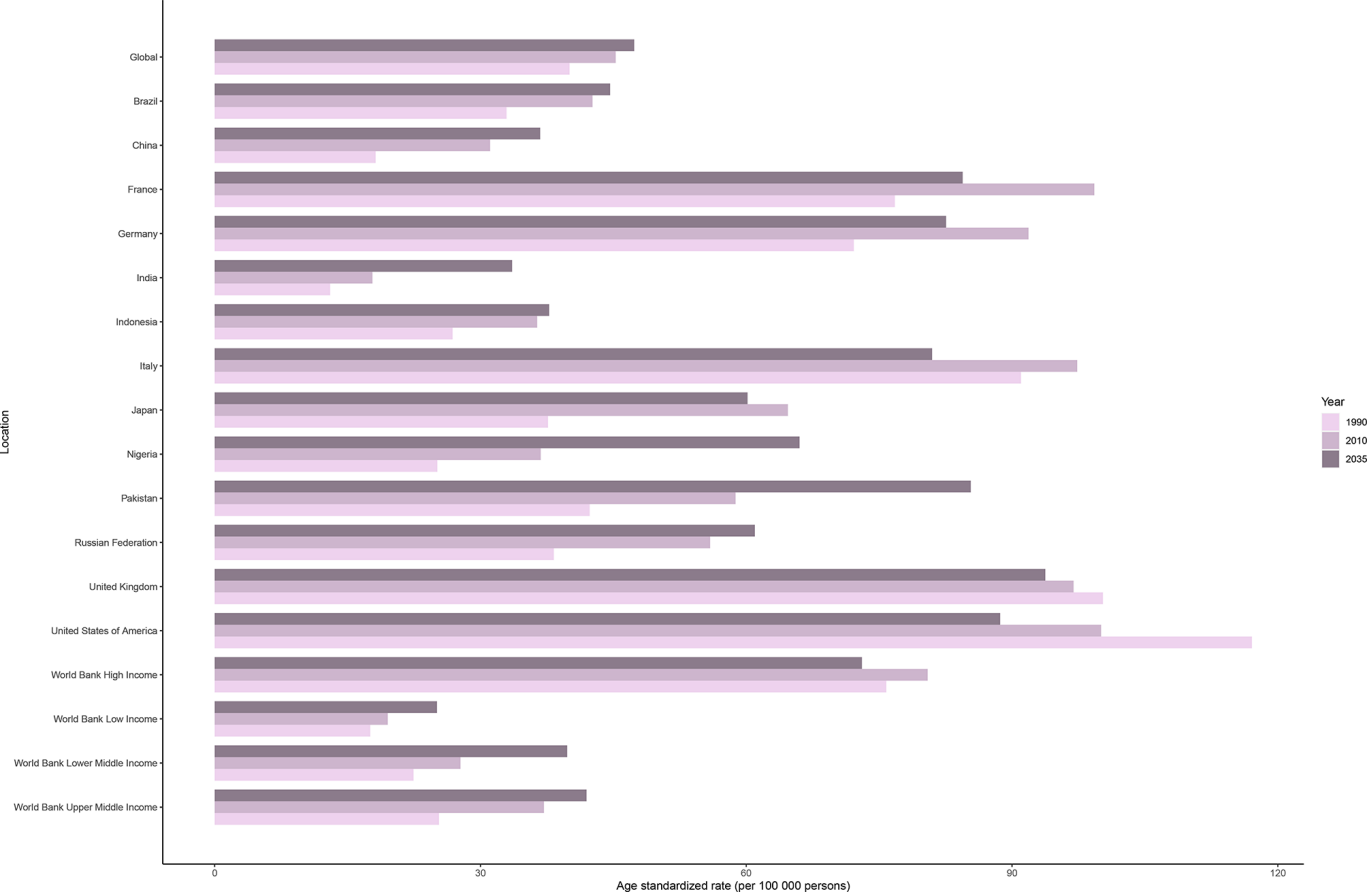
Age-standardized incidence rates of female breast cancer in 1990 and 2010, and predicted incidence rates in 2035 worldwide, in four World Bank income regions, and in 13 countries.

The incidence rate of female breast cancer was predicted to continue to increase globally, from 45.26 in 2010 to 47.36 in 2035. As shown in **Figure 5**, ASIRs in all but high-income regions were predicted to increase by 2035. Despite the projected decline, ASIR would still be significantly higher in the high-income regions than in the other three regions in 2035. Developed countries with relatively high ASIRs, such as the USA (100.06 in 2010 vs. 88.66 in 2035), the UK (96.95 in 2010 vs. 93.76 in 2035), and France (99.29 in 2010 vs. 84.43 in 2035), will show a downward trend. Meanwhile, developing countries with relatively low ASIRs, such as China (31.09 in 2010 vs. 36.75 in 2035), India (17.81 in 2010 vs. 33.58 in 2035), and Pakistan (58.79 in 2010 vs. 85.35 in 2035), will mostly show an increasing trend.

### Predictions by Age Groups

**Figure 6:**
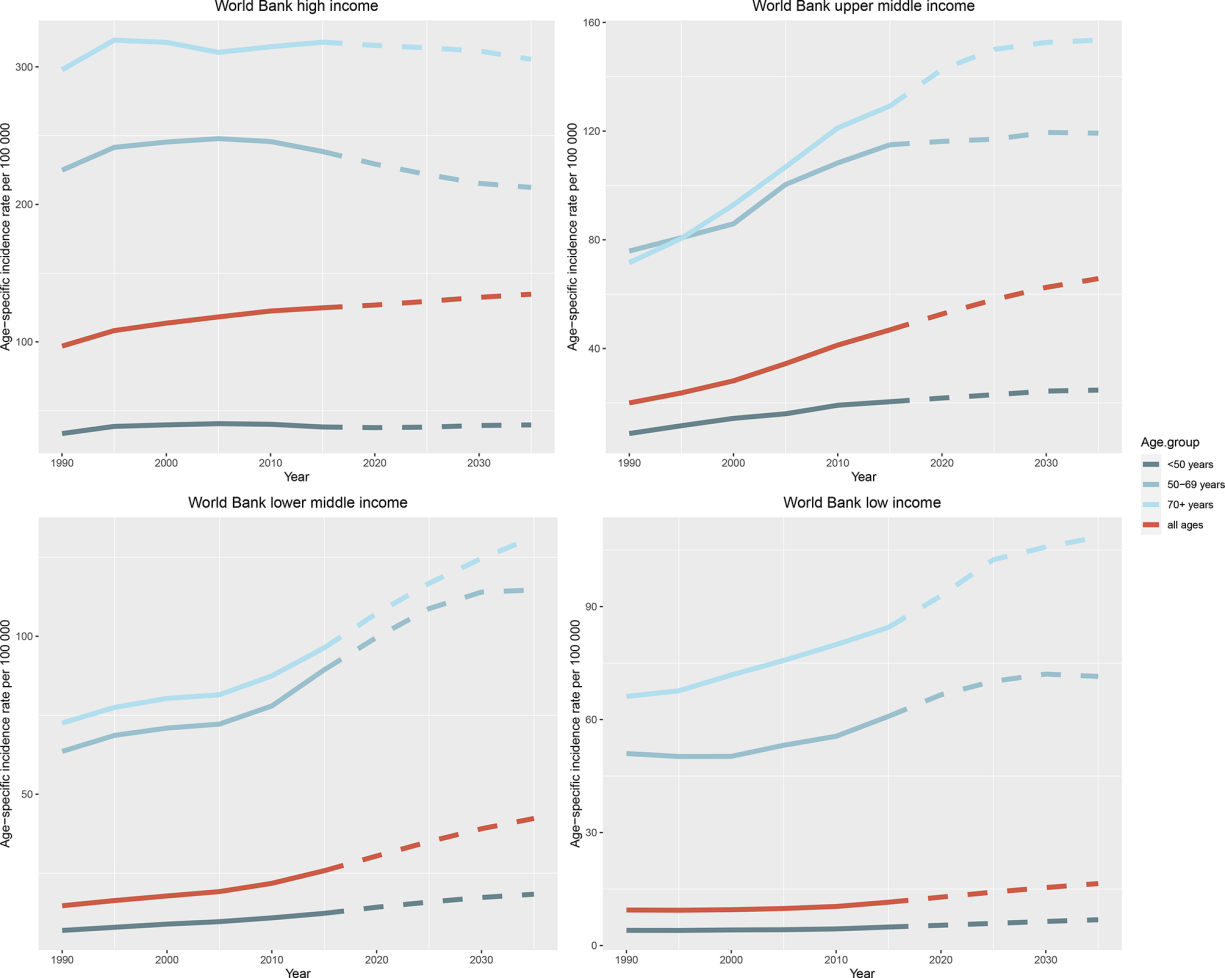
Trends in age-specific incidence rates of female breast cancer in four World Bank income regions by age group from 1990 to 2035. Note: Observed rates are plotted with solid lines and predicted rates are plotted with dashed lines.

The observed and predicted incidence trends differed among age groups (**Figure 6** and **Supplementary Figure 2**). In most countries and regions, the incidence of female breast cancer is the highest among those aged over 70 years and the lowest among those aged <50 years, and it will increase in all three age groups (< 50, 50 to 69, and > 70 years) until 2035. One exception is the high-income region, where age specific breast cancer incidence rates can be expected to decline significantly in people aged 50 to 69 years (245.70 per 100 000 person-year in 2010 vs. 212.36 per 100 000 person-year in 2035) and over 70 years (314.61 in 2010 vs. 305.49 in 2035), and the downward trend was not obvious in those under 50 years old (40.13 in 2010 vs. 39.67 in 2035). The projected trends for the UK and the USA are broadly similar, with a significant decline in the cohorts over 50 years old, and a slight increase in those younger than 50 years old. Japan is a unique case in that the incidence will decrease slightly in people under 50 years old, significantly decrease in people aged 50 to 69 years, and significantly increase in people over 70 years old until 2035. In Indonesia and the Russian Federation, the incidence is the highest in the 50–69 years age group and is predicted to remain stable until 2035.

## Discussion

Worldwide, the incident cases of female breast cancer and ASIRs showed annual increases, and the higher the SDI, the higher the incidence. According to our prediction, ASIRs will continue to increase in the future, except for the high-income regions; the global ASIR will reach 47.36 per 100 000 person-year in 2035. For most countries and regions, ASIRs will continue to be the highest among people aged over 70 years. However, in Indonesia and the Russian Federation, rates will remain the highest among people aged 50 to 69 years.

Of the six selected countries, only the USA showed a downward trend in ASIRs from 1990 to 2019, whereas India and China were the two countries where the ASIRs increased every year. ASIRs remained stable or declined slightly in the Russian Federation, Japan, and Germany over the past decade. However, despite the decline, the USA still had the highest ASIR among the six countries in 2019, whereas India had the lowest.

The USA’s success in breast cancer rates reduction was influenced by both period and cohort effects, especially among those born after 1960. The rise in breast cancer incidence rates in the early 1990s might reflect changes in women’s reproductive patterns at the time, such as delaying childbirth and declining fertility, both of which are associated with breast cancer risk (13). The increasing use of mammography screening was also a major factor, with screening rates rising from 29% in 1987 to 70% in 2000 (14). However, since the late 1990s, the USA’s ASIRs demonstrated a clear declining trend, especially for invasive breast cancer, which showed a sharp drop (nearly 13%) between 1999 and 2004 (15). This sharp drop could be attributed to the decline in menopausal hormone therapy after the publication of a randomized trial by the Women’s Health Initiative that linked menopausal hormone therapy with an increased risk of breast cancer (16). Another factor may be the slight decline in mammography screening rates since 2000 (17). Notably, the decrease in the incidence rates of breast cancer was largely confined to white women in the USA, mainly to estrogen-receptor positive subtypes (18). From 2012 to 2016, the incidence of hormone receptor (HR)-positive cancers gradually increased, whereas HR-negative tumors decreased by 1.5% to 2.6% each year (13). The reasons for this difference in trends remain unclear but may relate to changes in subtype-specific risk factors. For instance, parity is associated with a lower risk of HR-positive breast cancer but a higher risk of triple-negative breast cancer. meanwhile, the fertility rate in the USA declined from 118 births per 1,000 women in reproductive age to 69.4 in 2007, and then continued to decline to 60.3 in 2017 (19).

Germany recorded increased ASIRs from 1990 to 2009 and then showed a slow downward trend from 2010 to 2019, with little evidence of period and cohort effects. Changes in women’s reproductive behaviors in the 1990s (e.g., lower fertility rate and later childbearing) might be the major reason for the increase in breast cancer incidence, although this period was also marked by the increase in overweight and obesity rates among young German women (20). The national breast cancer screening program in Germany started in 2005 and was completely implemented nationwide until 2009 (21). A study compared the breast cancer incidence rates before the program began and 10 years later, and the results indicated that breast cancer incidence typically peaked with the introduction of mammography screening, driven mainly by an increase in early-stage breast cancer (21). In the long run, the increase in ASIRs after the introduction of the mammography screening program might indicate over-diagnosis, a highly concerning adverse consequence (22). The temporary reduction observed before 2006 may be due to a decline in the prescription of hormone replacement therapy (HRT), after two large studies (23, 24) reported that HRT increases the risk of breast cancer. According to the World Bank (25), the fertility rate in Germany began to show an upward trend from 2006, rising from 1.33 births per woman in 2006 to 1.57 in 2018. This increase may explain downward trend in the incidence of breast cancer in Germany after 2010, as infertility is a risk factor for the increased incidence of breast cancer.

Japan has unfavorable trends in breast cancer incidence, with significant period and cohort effects across 30 years. The ASIR peaked in women aged 45–49 years, consistent with the highest mammography screening rate in this age group, suggesting that a screening effect might play an important role in the increased incidence rate (26). Another possible explanation is that a general tendency toward later marriage and declining birth rates, as well as changes in the lifestyle of Japanese women, has led to an increase in the number of women at higher risk of breast cancer. Indeed, breast cancer incidence is on the rise as younger generations adopt Western culture, diet, and habits (27). A study by the Japan Public Health Center indicated that a Westernized dietary pattern (characterized by a high intake of red meat, refined grains, potatoes, and fat) is associated with an increased risk of breast cancer in Japanese women (28). Atomic bombing (29) and nuclear radiation (30) may also have contributed to the incidence rates, although these factors are more localized. In recent decades, the incidence in Japan had shown a declining trend. As mentioned before, the incidence in women aged 45–49 years was higher than that in women aged >50 years. With the aging of the population, the proportion of women over 50 years old increases, which might be one reason for the recent decreasing trend in ASIR (31).

The ASIR of female breast cancer in Russia continued to increase over the study period, rising sharply between 1990 and 1995, then falling back and then rising again at a slower rate. The increasing incidence could partially be explained by changing fertility rates in Russia (32), which fell sharply in the early 20^th^ century and reached its lowest point in the 1950s. In the post-World War II generations, the incidence was relatively stable, which could be attributed to several balancing factors, including a decline in fertility, increase in obesity prevalence, and changes in dietary habits, as well as improved diagnostic methods and accidental early detection (32). Environmental factors, such as radiation, may also be at play. A study on the Techa River cohort (suffered from radioactive wastes) reported a significant temporal trend of breast cancer incidence, characterized by a linear dose-response relation (33). The fallout from ionizing radiation has also been significant, with women living in the Bryansk Oblast experiencing a significantly increased risk of breast cancer in the decades after the Chernobyl accident (34). A related cohort study found a dose-dependent relation between radiation and breast cancer incidence, and younger women are identified as at a higher risk when exposed.

China showed increasing ASIRs for the study period, driven by significant period and cohort effects. Higher incidence rates were observed among older women and more recent birth cohorts. These trends could be attributed to changes in lifestyle patterns, such as dietary change, decreased physical activity, and increased obesity rates (35). Data from the National Chronic Disease and Risk Factor Surveillance showed that the overall prevalence of obesity in Chinese adults tripled from 2004 to 2014 (14.0%) (36). The increasing obesity rate is reported as a consequence of nutrition transitions and changes in physical activity. Reproductive patterns were also likely to contribute to the ASIR increase in breast cancer; China implemented the one-child policy in the 1970s. Compared with older generations, younger women reported earlier ages at menarche, later ages at menopause, delayed childbearing, lower fertility rates, and less breastfeeding. According to a large-scale cohort study comparing women born in the 1930s and 1970s in China, the age of menarche was 1.8 years earlier (16.1 vs. 14.3), age of first birth was 6 years later (urban: 19.0 vs. 25.9; rural: 18.3 vs. 23.8), breastfeeding duration was 4 to 5 months shorter (urban: 16 vs. 11; rural: 18 vs. 14), and menopause was 1.4 years later (47.9 vs. 49.3) in the younger cohort (37). Another factor that should not be ignored is the increase in breast cancer screening rates. There has been a marked improvement in the number and effectiveness of national cancer registries between 2003 and 2011—the number of registries rose from 35 to 243, and the covered population increased from 4.34% to 13.01%—as well as an improvement in the quality of data collected (38).

India had the lowest ASIR of breast cancer among the six selected countries, although the ASIR in India continued to rise, influenced by period and cohort effects. The change in trend might be explained by lifestyle changes *via* economic liberalization since 1991 (39), and period-specific declines were observed in more recent generations. The increasing trend among older women might be related to diagnostic improvement or increased awareness of the disease, including improved diagnosis and self-referral for screening, which have led to a higher average risk of breast cancer diagnosis (40). The increase in incidence could also be partially owing to changes in risk factors, including reproductive, dietary, and other lifestyle patterns associated with economic growth (41). In the case of reproductive factors, the marriage rate of Indian women by the age of 18 years declined from 54.2% in 1992–1993 to 44.5% in 2005–2006, parity declined from 3.39 live-born children per women to 2.26, and contraceptive use increased from 1.2% to 3.1% during the same period (39). The incidence rates, education level, and income are higher in urban areas compared with rural areas, suggesting that access to care and use of preventive care might be issues that affect cancer incidence (42). Other epidemiological studies have suggested that obese or overweight women are at a higher risk of breast cancer (43). According to the India National Family Health Survey (44), the percentage of married women aged 19–49 years who were overweight or obese increased from 11% in 1995–1996 to 18% in 2015–2016, which may also contribute to the increase in breast cancer incidence.

Based on the prediction of the breast cancer incidence in women worldwide, across four regions with different income levels, and in several countries, the global incidence of breast cancer will continue to increase, and the change pattern presents a certain correlation with economic development. Developed countries, such as the USA, have a relatively high incidence, although incidence has started and will continue to decline in the future, especially in middle-aged and older women. Developing countries, such as India, have a relatively low incidence but it will continue to increase in the future, and will be higher than that of some of the developed countries. Therefore, for developing countries, attention should be paid to the prevention and treatment of breast cancer to control further increases in incidence. Non-genetic, modifiable risk factors should be highlighted in these regions and countries. Income levels, education, and insurance status, which might affect access to health care and influence breast cancer stage at diagnosis, should also be improved. Other measures include changing reproductive patterns, such as having a first child at a younger age, increasing parity, and extending breastfeeding for one or more years; reducing menopausal HRT use; and promoting lifestyle changes, such as reducing smoking and drinking alcohol, exercising regularly, eating a healthy diet, and staying in shape (45). Developed countries, where breast cancer incidence has shown a downward trend, already have a fairly sophisticated strategy for prevention, such as chemical and surgical prophylaxis for people with genetic risk factors (46). If the downward trend continues, these countries will complete the transition from high incidence and low mortality to low incidence and low mortality.

Our study has several strengths and limitations. First, to our knowledge, this work is the first attempt to compare and analyze breast cancer incidence in multiple countries and to predict the incidence at the national, regional, and global levels. However, owing to the limitations of the GBD database (2), we could not obtain the original data to conduct more accurate analyses, particularly detailed analyses accounting for the clinical classification of breast cancer. Second, although the APC model was applied to analyze the morbidity of female breast cancer based on GBD 1990–2019, this GBD-based study is not a cohort study. Therefore, large-scale cohort studies in different countries are strongly needed to establish location- and time-specific RRs. Third, predictions based on current incidence trends are based on the assumption that trends observed in the past will continue in the future, thereby ignoring the impact of risk factor prevalence and trends; thus, our predictions involved a degree of uncertainty (47). Nonetheless, when comparing our predicted data with the published data, the two are remarkably close, which proves that our prediction is relatively accurate (Supplementary).

## Conclusions

The global burden of female breast cancer continued to increase from 1990 to 2019, especially in developing countries. Among the six selected countries, only the USA showed a declining trend during the study period, whereas the other five demonstrated increased or stable trends. From the perspective of age, the peak age of onset in developed countries was later compared with developing countries, at 60–70 years old. Moreover, the results of the six countries showed different degrees of period and cohort effects. In China, the incidence rate increased rapidly. According to predictions, by 2035, the incidence of global female breast cancer is expected to increase further. Among the four regions divided by income level, only high-income regions will see a decline in incidence rates. Therefore, developing countries and regions should pay more attention to the prevention of breast cancer, strengthen the public’s awareness of breast cancer risk factors, and formulate corresponding policies to achieve better prevention of breast cancer. Regions and countries where breast cancer incidence are predicted to rise should do more in terms of prevention and improve the efficiency of screening, in order to reduce the future burden.

## Data Availability Statement

The original contributions presented in the study are included in the article/**Supplementary Material**. Further inquiries can be directed to the corresponding authors.

## Ethics Statement

For GBD study, a waiver of informed consent was reviewed and approved by the Institutional Review Board of the University of Washington.

## Author Contributions

YL, JZ, YD, HK, and ZD designed the study. YL, XD, WL, BW, and DX analyzed the data and performed the statistical analyses. YL, JZ, JH, YZ, PX, JY, ZZ, LZ, SY, and YW drafted the initial manuscript. All authors reviewed the drafted manuscript for critical content and approved the final version. The corresponding author attests that all listed authors meet authorship criteria and that no others meeting the criteria have been omitted.

## Funding

Natural Science Foundation of Zhejiang Province (LZ22H160005).

## Conflict of Interest

The authors declare that the research was conducted in the absence of any commercial or financial relationships that could be construed as a potential conflict of interest.

## Publisher’s Note

All claims expressed in this article are solely those of the authors and do not necessarily represent those of their affiliated organizations, or those of the publisher, the editors and the reviewers. Any product that may be evaluated in this article, or claim that may be made by its manufacturer, is not guaranteed or endorsed by the publisher.

## Abbreviations

APC, age-period-cohort; ASR, age standardized rate; ASIR, age standardized incidence rate; ASDR, age standardized death rate; GBD, Global Burden of Disease; HR, hormone receptor; HRT, hormone replacement therapy; RR, relative risk; SDI, sociodemographic index; UN, United Nation; UK, United Kingdom; USA, United States of America; WB, World Bank.
