# Supplementary material for: Global Burden of Female Breast Cancer: Age-Period-Cohort Analysis of Incidence Trends From 1990 to 2019 and Forecasts for 2035
Source: Front Oncol. 2022 Jun 9;12:891824. doi: 10.3389/fonc.2022.891824 (PMC9218744; doi:10.3389/fonc.2022.891824)

**Global Burden of Female Breast Cancer: Age-Period-Cohort Analysis of Incidence Trends from 1990 to 2019 and Forecasts for 2035**

Yizhen Li^1,2,†^, Jinxin Zheng^1,†^, Yujiao Deng^1,2,†^, Xinyue Deng^1^, Weiyang Lou^1^, Bajin Wei^1^, Dong Xiang^3^, Jingjing Hu^4^, Yi Zheng^1,2^, Peng Xu^2^, Jia Yao^1^, Zhen Zhai^1,2^, Linghui Zhou^1^, Si Yang^1,2^, Ying Wu^1,2^, Huafeng Kang^2^, and Zhijun Dai^1,2^

Table of contents

Supplementary Figure 1 2

Supplementary Figure 2 3

Supplementary Figure 3 4

Supplementary Table 1 5

Supplementary Table 2 6

Model Validation 9

**Supplementary Figure 1.** Trends of age-standardized incidence rates for female breast cancer in six most burdensome countries from 1990 to 2019.


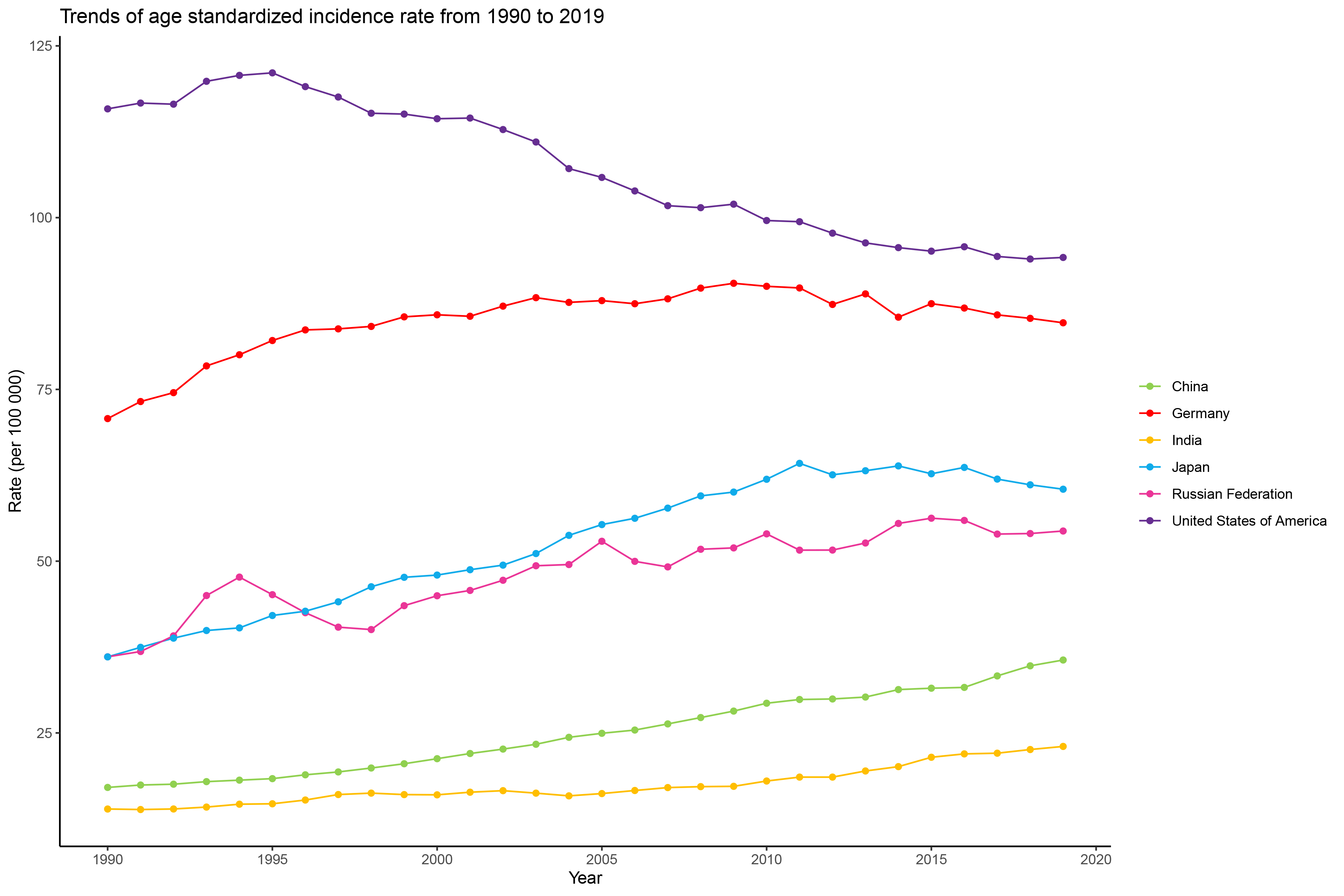


**Supplementary Figure 2.** Trends in age-specific incidence rates of female breast cancer in six selected most burdensome countries by age group from 1990 to 2035. Note: Observed rates are plotted with solid lines and predicted rates are plotted with dashed lines.


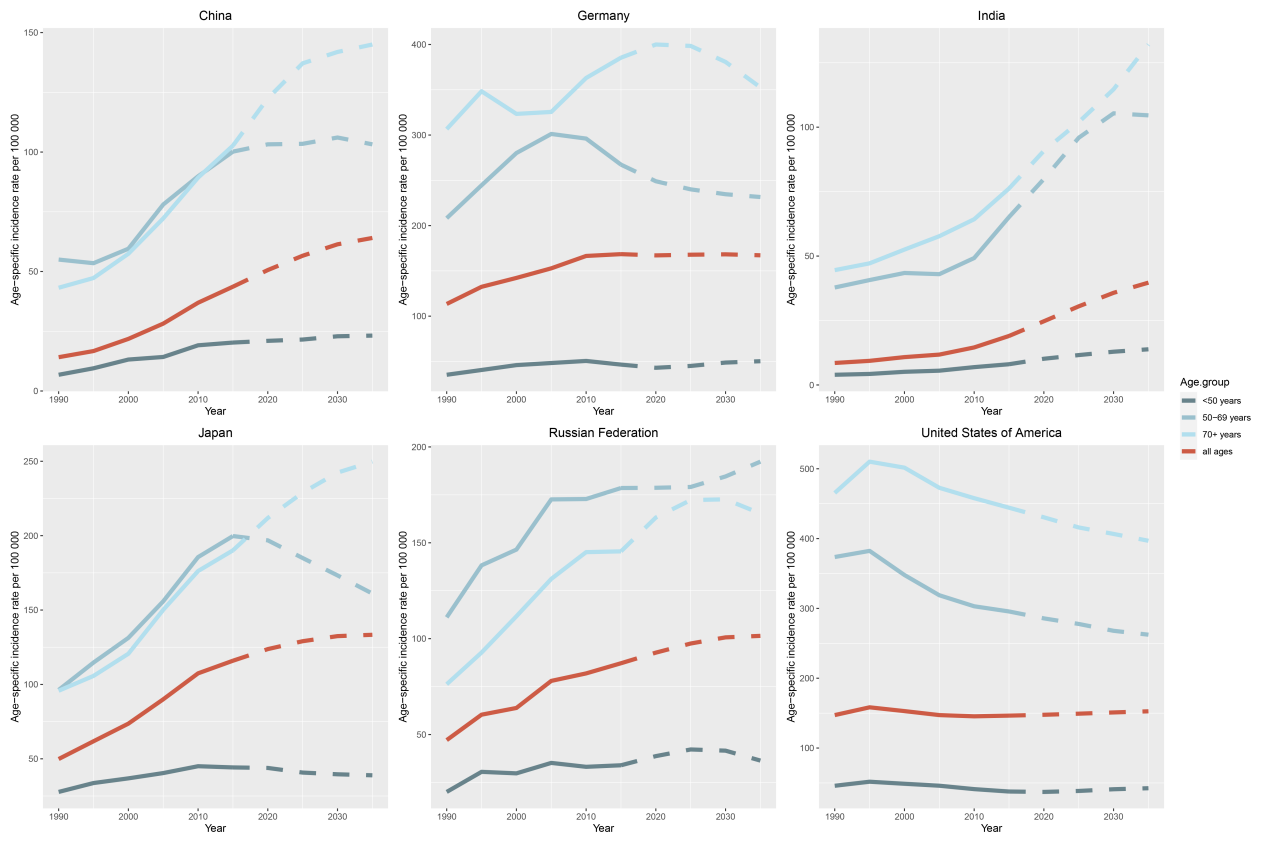


**Supplementary Figure 3.** Trends in age-specific incidence rates of female breast cancer worldwide and in 13 selected countries by age group from 1990 to 2035. Note: Observed rates are plotted with solid lines and predicted rates are plotted with dashed lines.


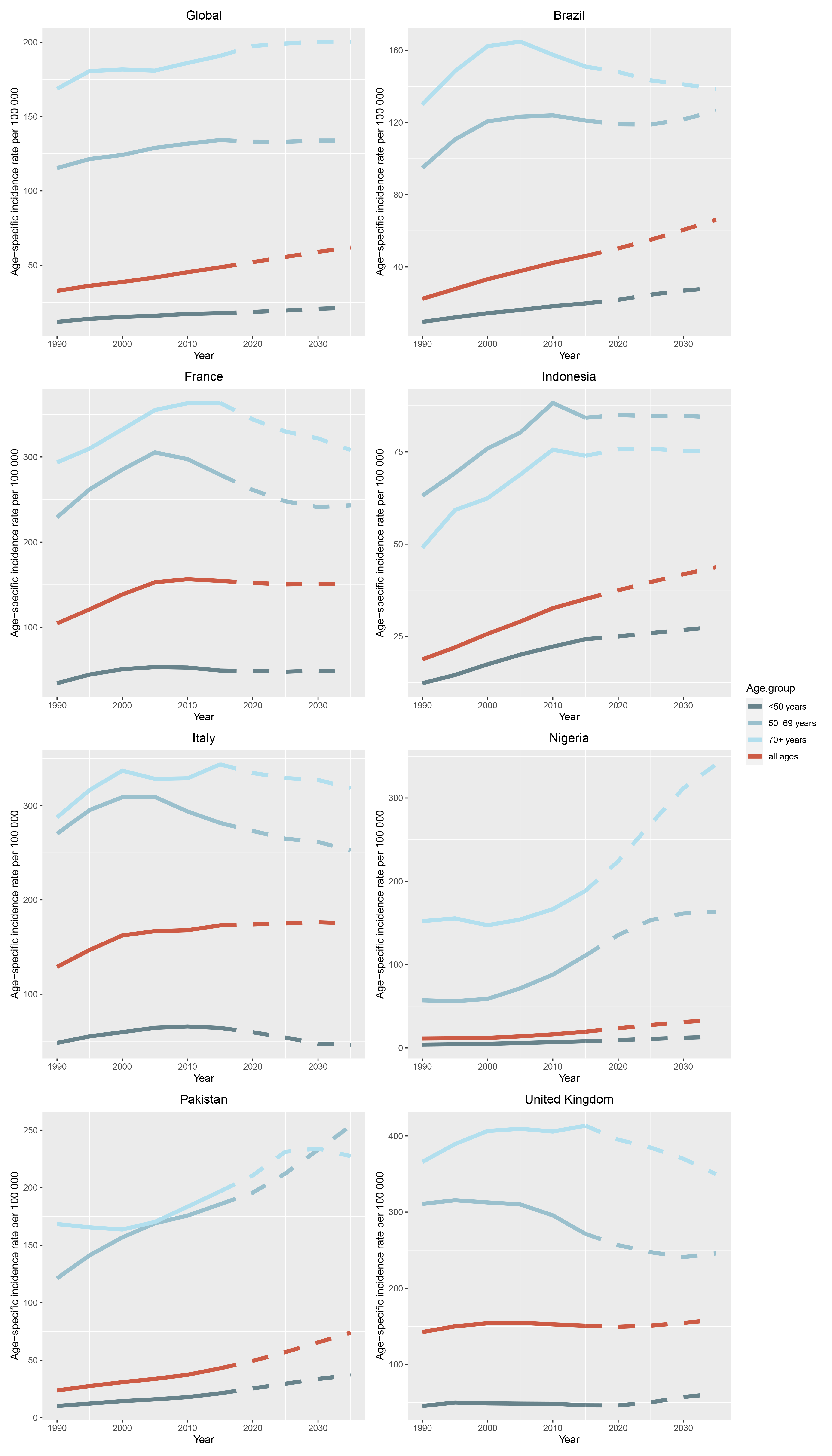


**Supplementary Table 1.** The ten countries with the highest number of incidence, deaths, prevalence, and disability-adjusted life years.

| **Rank** | **Incidence** | | | **Deaths** | | | **Prevalence** | | | **DALYs** | | |
| --- | --- | --- | --- | --- | --- | --- | --- | --- | --- | --- | --- | --- |
|  | **Country** | **Cases** | **Population (thousands)** | **Country** | **Cases** | **Population (thousands)** | **Country** | **Cases** | **Population (thousands)** | **Country** | **Cases** | **Population (thousands)** |
| **1** | China | 368 375 | 1 433 784* | China | 93 499 | 1 433 784* | China | 3 473 262 | 1 433 784* | China | 2 877 240 | 1 433 784* |
| **2** | USA | 251 531 | 329 065 | India | 82 099 | 1 366 418 | USA | 2 787 997 | 329 065 | India | 2 659 243 | 1 366 418 |
| **3** | India | 144 086 | 1 366 418 | USA | 54 402 | 329 065 | India | 1 106 547 | 1 366 418 | USA | 1 387 670 | 329 065 |
| **4** | Japan | 74 260 | 126 860 | Pakistan | 31 177 | 216 565 | Japan | 884 138 | 126 860 | Pakistan | 1 096 288 | 216 565 |
| **5** | Germany | 69 662 | 83 517 | Indonesia | 26 166 | 270 626 | Germany | 760 184 | 83 517 | Indonesia | 973 809 | 270 626 |
| **6** | Russian Federation | 67 692 | 145 872 | Russian Federation | 23 486 | 145 872 | Russian Federation | 662 018 | 145 872 | Russian Federation | 636 320 | 145 872 |
| **7** | Italy | 53 095 | 60 550 | Germany | 21 486 | 83 517 | Italy | 621 334 | 60 550 | Brazil | 588 448 | 211 050 |
| **8** | UK | 52 730 | 67 530 | Brazil | 19 764 | 211 050 | France | 582 682 | 65 130 | Germany | 467 068 | 83 517 |
| **9** | Brazil | 51 856 | 211 050 | Japan | 15 911 | 126 860 | UK | 570 689 | 67 530 | Nigeria | 435 400 | 200 964 |
| **10** | France | 50 715 | 65 130 | UK | 15 162 | 67 530 | Brazil | 437 220 | 211 050 | Japan | 395 292 | 126 860 |

The data included in this table were obtained from GBD 2019 and UN population prospects 2019 revision. * indicated that, according to the UN Population Divisions, the population of China is counted in four parts, including China, Hong Kong SAR, Macao SAR, and Taiwan Province, and the population number listed in this table is the first part. Abbreviations: disability-adjusted life years, DALYs; Global Burden of Disease, GBD; special administrative region, SAR; United Nation, UN; the United States of America, USA; the United Kingdom, UK.

**Supplementary Table 2.** Incidence rates of female breast cancer by selected age group, period, and birth cohorts in six countries, 1990-2019.

| **Period** | **20 to 24** | **25 to 29** | **30 to 34** | **35 to 39** | **40 to 44** | **45 to 49** | **50 to 54** | **55 to 59** | **60 to 64** | **65 to 69** | **70 to 74** | **75 to 79** | **Birth Cohort** |
| --- | --- | --- | --- | --- | --- | --- | --- | --- | --- | --- | --- | --- | --- |
| **China** |  |  |  |  |  |  |  |  |  |  |  | 45.1 | 1908 to 1912 |
|  |  |  |  |  |  |  |  |  |  |  | 46.8 | 47.9 | 1913 to 1917 |
|  |  |  |  |  |  |  |  |  |  | 51.4 | 51.9 | 58.6 | 1918 to 1922 |
|  |  |  |  |  |  |  |  |  | 53.7 | 54.3 | 62.8 | 70.6 | 1923 to 1927 |
|  |  |  |  |  |  |  |  | 58.3 | 54.5 | 68.6 | 76.5 | 86.6 | 1928 to 1932 |
|  |  |  |  |  |  |  | 51.2 | 50.4 | 66.7 | 81.8 | 90.6 | 99.8 | 1933 to 1937 |
|  |  |  |  |  |  | 41.2 | 53.3 | 65.7 | 83.2 | 100.3 | 105.2 |  | 1938 to 1942 |
|  |  |  |  |  | 31.4 | 56.2 | 64.5 | 84.7 | 101 | 116 |  |  | 1943 to 1947 |
|  |  |  |  | 18.1 | 34.9 | 59.2 | 78 | 92.7 | 111.7 |  |  |  | 1948 to 1952 |
|  |  |  | 7.4 | 20.5 | 40.7 | 61.6 | 81.5 | 104.7 |  |  |  |  | 1953 to 1957 |
|  |  | 2.3 | 11.5 | 22 | 46.2 | 67.5 | 87.8 |  |  |  |  |  | 1958 to 1962 |
|  | 0.9 | 4.1 | 11.7 | 24.3 | 49.5 | 69.2 |  |  |  |  |  |  | 1963 to 1967 |
| 1990 to 1994 | 1.3 | 4.5 | 10.8 | 26.6 | 55.5 |  |  |  |  |  |  |  | 1968 to 1972 |
| 1995 to 1999 | 1.4 | 4.2 | 14.5 | 31.8 |  |  |  |  |  |  |  |  | 1973 to 1977 |
| 2000 to 2004 | 1.3 | 5.6 | 16 |  |  |  |  |  |  |  |  |  | 1978 to 1982 |
| 2005 to 2009 | 1.6 | 5.8 |  |  |  |  |  |  |  |  |  |  | 1983 to 1987 |
| 2010 to 2014 | 1.8 |  |  |  |  |  |  |  |  |  |  |  | 1988 to 1992 |
| 2015 to 2019 |  |  |  |  |  |  |  |  |  |  |  |  |  |
| **Germany** |  |  |  |  |  |  |  |  |  |  |  | 338.1 | 1908 to 1912 |
|  |  |  |  |  |  |  |  |  |  |  | 276.1 | 309.5 | 1913 to 1917 |
|  |  |  |  |  |  |  |  |  |  | 266.6 | 318.5 | 309 | 1918 to 1922 |
|  |  |  |  |  |  |  |  |  | 237.4 | 283.6 | 289.8 | 310.6 | 1923 to 1927 |
|  |  |  |  |  |  |  |  | 190.1 | 265.5 | 315.9 | 315 | 346.3 | 1928 to 1932 |
|  |  |  |  |  |  |  | 183.5 | 249.2 | 315.9 | 369.5 | 348.3 | 374.7 | 1933 to 1937 |
|  |  |  |  |  |  | 161.7 | 233.7 | 287.6 | 350 | 386.5 | 369.2 |  | 1938 to 1942 |
|  |  |  |  |  | 107.3 | 173.2 | 229.9 | 268.3 | 312.3 | 349.6 |  |  | 1943 to 1947 |
|  |  |  |  | 54.7 | 113.8 | 182.7 | 216.8 | 242.7 | 287.7 |  |  |  | 1948 to 1952 |
|  |  |  | 26.6 | 57 | 115.1 | 171.3 | 198.5 | 228.3 |  |  |  |  | 1953 to 1957 |
|  |  | 7.6 | 27.5 | 55.1 | 108.9 | 161.4 | 191.2 |  |  |  |  |  | 1958 to 1962 |
|  | 1.2 | 8.3 | 26.7 | 56.4 | 112.2 | 161.2 |  |  |  |  |  |  | 1963 to 1967 |
| 1990 to 1994 | 1.4 | 7.6 | 25.3 | 57.6 | 111.6 |  |  |  |  |  |  |  | 1968 to 1972 |
| 1995 to 1999 | 1.5 | 8.1 | 27 | 56.9 |  |  |  |  |  |  |  |  | 1973 to 1977 |
| 2000 to 2004 | 1.4 | 9 | 28.7 |  |  |  |  |  |  |  |  |  | 1978 to 1982 |
| 2005 to 2009 | 1.4 | 9.6 |  |  |  |  |  |  |  |  |  |  | 1983 to 1987 |
| 2010 to 2014 | 1.5 |  |  |  |  |  |  |  |  |  |  |  | 1988 to 1992 |
| 2015 to 2019 |  |  |  |  |  |  |  |  |  |  |  |  |  |
| **India** |  |  |  |  |  |  |  |  |  |  |  | 55 | 1908 to 1912 |
|  |  |  |  |  |  |  |  |  |  |  | 52.2 | 59.2 | 1913 to 1917 |
|  |  |  |  |  |  |  |  |  |  | 48.6 | 57.3 | 64.2 | 1918 to 1922 |
|  |  |  |  |  |  |  |  |  | 41.7 | 53 | 61.5 | 66.8 | 1923 to 1927 |
|  |  |  |  |  |  |  |  | 41.8 | 48.4 | 56.2 | 61.9 | 63.6 | 1928 to 1932 |
|  |  |  |  |  |  |  | 36.5 | 47.7 | 47.8 | 56.1 | 67.2 | 69.2 | 1933 to 1937 |
|  |  |  |  |  |  | 26.1 | 46.1 | 49.3 | 54.4 | 67.1 | 73.6 |  | 1938 to 1942 |
|  |  |  |  |  | 19 | 32.9 | 42.1 | 49.8 | 59.2 | 69.7 |  |  | 1943 to 1947 |
|  |  |  |  | 11.6 | 21.7 | 34.1 | 41 | 55.4 | 68.2 |  |  |  | 1948 to 1952 |
|  |  |  | 5.6 | 12.2 | 22.2 | 33.7 | 48.1 | 69.6 |  |  |  |  | 1953 to 1957 |
|  |  | 2.5 | 6.5 | 14.9 | 24.2 | 39 | 71.8 |  |  |  |  |  | 1958 to 1962 |
|  | 1.1 | 2.9 | 6.7 | 14.7 | 25.8 | 43.5 |  |  |  |  |  |  | 1963 to 1967 |
| 1990 to 1994 | 1.2 | 3.3 | 7.1 | 15.6 | 32 |  |  |  |  |  |  |  | 1968 to 1972 |
| 1995 to 1999 | 1.3 | 3.2 | 7.5 | 17.3 |  |  |  |  |  |  |  |  | 1973 to 1977 |
| 2000 to 2004 | 1.4 | 4 | 8.1 |  |  |  |  |  |  |  |  |  | 1978 to 1982 |
| 2005 to 2009 | 1.6 | 4 |  |  |  |  |  |  |  |  |  |  | 1983 to 1987 |
| 2010 to 2014 | 1.6 |  |  |  |  |  |  |  |  |  |  |  | 1988 to 1992 |
| 2015 to 2019 |  |  |  |  |  |  |  |  |  |  |  |  |  |
| **Japan** |  |  |  |  |  |  |  |  |  |  |  | 99.3 | 1908 to 1912 |
|  |  |  |  |  |  |  |  |  |  |  | 98.3 | 106.8 | 1913 to 1917 |
|  |  |  |  |  |  |  |  |  |  | 104.2 | 110.4 | 131 | 1918 to 1922 |
|  |  |  |  |  |  |  |  |  | 103.3 | 118.2 | 133.4 | 165.9 | 1923 to 1927 |
|  |  |  |  |  |  |  |  | 104.1 | 118.4 | 136.1 | 166.5 | 191.8 | 1928 to 1932 |
|  |  |  |  |  |  |  | 103 | 114.1 | 136 | 166.3 | 188 | 191.6 | 1933 to 1937 |
|  |  |  |  |  |  | 108.7 | 122.6 | 133 | 170.3 | 204.5 | 194.4 |  | 1938 to 1942 |
|  |  |  |  |  | 84.3 | 133.6 | 137.9 | 162 | 203.4 | 217.9 |  |  | 1943 to 1947 |
|  |  |  |  | 46.3 | 95.2 | 148.6 | 159.8 | 179.6 | 208.4 |  |  |  | 1948 to 1952 |
|  |  |  | 21.1 | 51.1 | 99.9 | 166.5 | 174 | 180.8 |  |  |  |  | 1953 to 1957 |
|  |  | 6 | 22.4 | 53.5 | 110.1 | 162.6 | 174.1 |  |  |  |  |  | 1958 to 1962 |
|  | 1.1 | 6.2 | 22.5 | 55.1 | 106.5 | 151.1 |  |  |  |  |  |  | 1963 to 1967 |
| 1990 to 1994 | 1.3 | 6.4 | 23.5 | 53.3 | 99.1 |  |  |  |  |  |  |  | 1968 to 1972 |
| 1995 to 1999 | 1.5 | 7.6 | 24.7 | 49 |  |  |  |  |  |  |  |  | 1973 to 1977 |
| 2000 to 2004 | 1.8 | 8.1 | 23.4 |  |  |  |  |  |  |  |  |  | 1978 to 1982 |
| 2005 to 2009 | 1.7 | 7.9 |  |  |  |  |  |  |  |  |  |  | 1983 to 1987 |
| 2010 to 2014 | 1.6 |  |  |  |  |  |  |  |  |  |  |  | 1988 to 1992 |
| 2015 to 2019 |  |  |  |  |  |  |  |  |  |  |  |  |  |
| **Russian Federation** |  |  |  |  |  |  |  |  |  |  |  | 82.4 | 1908 to 1912 |
|  |  |  |  |  |  |  |  |  |  |  | 100.1 | 98.2 | 1913 to 1917 |
|  |  |  |  |  |  |  |  |  |  | 115.6 | 111.6 | 119.5 | 1918 to 1922 |
|  |  |  |  |  |  |  |  |  | 115.5 | 119.8 | 128.9 | 130.9 | 1923 to 1927 |
|  |  |  |  |  |  |  |  | 116 | 128.4 | 145.3 | 144.6 | 143.7 | 1928 to 1932 |
|  |  |  |  |  |  |  | 116.5 | 127.9 | 163.8 | 167.9 | 174.2 | 160.5 | 1933 to 1937 |
|  |  |  |  |  |  | 113.8 | 123.9 | 157.5 | 168.9 | 180 | 183.1 |  | 1938 to 1942 |
|  |  |  |  |  | 80 | 110.5 | 146.6 | 158.4 | 179.2 | 192.7 |  |  | 1943 to 1947 |
|  |  |  |  | 40.1 | 72.6 | 121 | 140.3 | 164.9 | 186.6 |  |  |  | 1948 to 1952 |
|  |  |  | 16.9 | 35.9 | 77 | 117.3 | 138.4 | 165.9 |  |  |  |  | 1953 to 1957 |
|  |  | 5.1 | 15.6 | 38.9 | 80.5 | 120.3 | 145.5 |  |  |  |  |  | 1958 to 1962 |
|  | 1 | 4.9 | 17.2 | 42.9 | 81.4 | 124.5 |  |  |  |  |  |  | 1963 to 1967 |
| 1990 to 1994 | 1.1 | 5.7 | 21.3 | 45.9 | 88.5 |  |  |  |  |  |  |  | 1968 to 1972 |
| 1995 to 1999 | 1.2 | 7.4 | 23 | 49.3 |  |  |  |  |  |  |  |  | 1973 to 1977 |
| 2000 to 2004 | 1.2 | 6.9 | 21.7 |  |  |  |  |  |  |  |  |  | 1978 to 1982 |
| 2005 to 2009 | 1.2 | 6.2 |  |  |  |  |  |  |  |  |  |  | 1983 to 1987 |
| 2010 to 2014 | 1.1 |  |  |  |  |  |  |  |  |  |  |  | 1988 to 1992 |
| 2015 to 2019 |  |  |  |  |  |  |  |  |  |  |  |  |  |
| **United States of America** |  |  |  |  |  |  |  |  |  |  |  | 503.3 | 1908 to 1912 |
|  |  |  |  |  |  |  |  |  |  |  | 496.8 | 517.9 | 1913 to 1917 |
|  |  |  |  |  |  |  |  |  |  | 483.4 | 504.9 | 508.6 | 1918 to 1922 |
|  |  |  |  |  |  |  |  |  | 411 | 475.5 | 481.7 | 467.4 | 1923 to 1927 |
|  |  |  |  |  |  |  |  | 331.1 | 409.4 | 458.8 | 439 | 462.4 | 1928 to 1932 |
|  |  |  |  |  |  |  | 279.7 | 338.8 | 399.9 | 430.3 | 429 | 432.5 | 1933 to 1937 |
|  |  |  |  |  |  | 232.1 | 284.8 | 326.2 | 367.6 | 419.3 | 419.4 |  | 1938 to 1942 |
|  |  |  |  |  | 149.2 | 225.7 | 260.6 | 282.2 | 339.4 | 380.1 |  |  | 1943 to 1947 |
|  |  |  |  | 73.4 | 147.6 | 210.8 | 228.6 | 266.7 | 339.1 |  |  |  | 1948 to 1952 |
|  |  |  | 31.9 | 72.3 | 139.3 | 186.1 | 218.4 | 253 |  |  |  |  | 1953 to 1957 |
|  |  | 9.8 | 32.6 | 68.9 | 122.8 | 175 | 207.3 |  |  |  |  |  | 1958 to 1962 |
|  | 1.7 | 9.8 | 30.3 | 58.6 | 115.3 | 161.4 |  |  |  |  |  |  | 1963 to 1967 |
| 1990 to 1994 | 1.9 | 9 | 26.6 | 56.3 | 119.9 |  |  |  |  |  |  |  | 1968 to 1972 |
| 1995 to 1999 | 2 | 8.8 | 26.5 | 56.8 |  |  |  |  |  |  |  |  | 1973 to 1977 |
| 2000 to 2004 | 2 | 8.7 | 30.4 |  |  |  |  |  |  |  |  |  | 1978 to 1982 |
| 2005 to 2009 | 1.8 | 9.6 |  |  |  |  |  |  |  |  |  |  | 1983 to 1987 |
| 2010 to 2014 | 1.7 |  |  |  |  |  |  |  |  |  |  |  | 1988 to 1992 |
| 2015 to 2019 |  |  |  |  |  |  |  |  |  |  |  |  |  |

The cohort follows from lower left to upper right, going diagonally.

**Model validation**

NORDPRED model (***Møller B, Fekjaer H, Hakulinen T, Sigvaldason H, Storm HH, Talback M, Haldorsen T (2003) Prediction of cancer incidence in the Nordic countries: empirical comparison of different approaches. Stat Med 22(17): 2751–2766***) was chosed to predict the future incidence rates of breast cancer in this manuscript, and it is a modified version of the age-period-cohort (APC) model. Predictions of the future cancer burden can be calculated by applying population forecasts to projections of cancer rates, while cancer rates are projected using the assumption that current trends continue into the future.

(***https://www.nature.com/articles/nrc1781***)

Therefore, the projected rates are based on the assumption that past trends will continue into the future, which APC hypothesis the temporal trends are stable over time. (***https://pubmed.ncbi.nlm.nih.gov/25146089/***)

Taking the global breast cancer incidence prediction data as an example, we have verified the predicted breast cancer incidence rates, and the results are shown in the figure below. Our model shows that the annual percentage change of the expected age-adjusted rates is contrast to no change over time ( *p*-value for recent < 0.001), which assumes that the current trend in the incidence rates continues into the future.


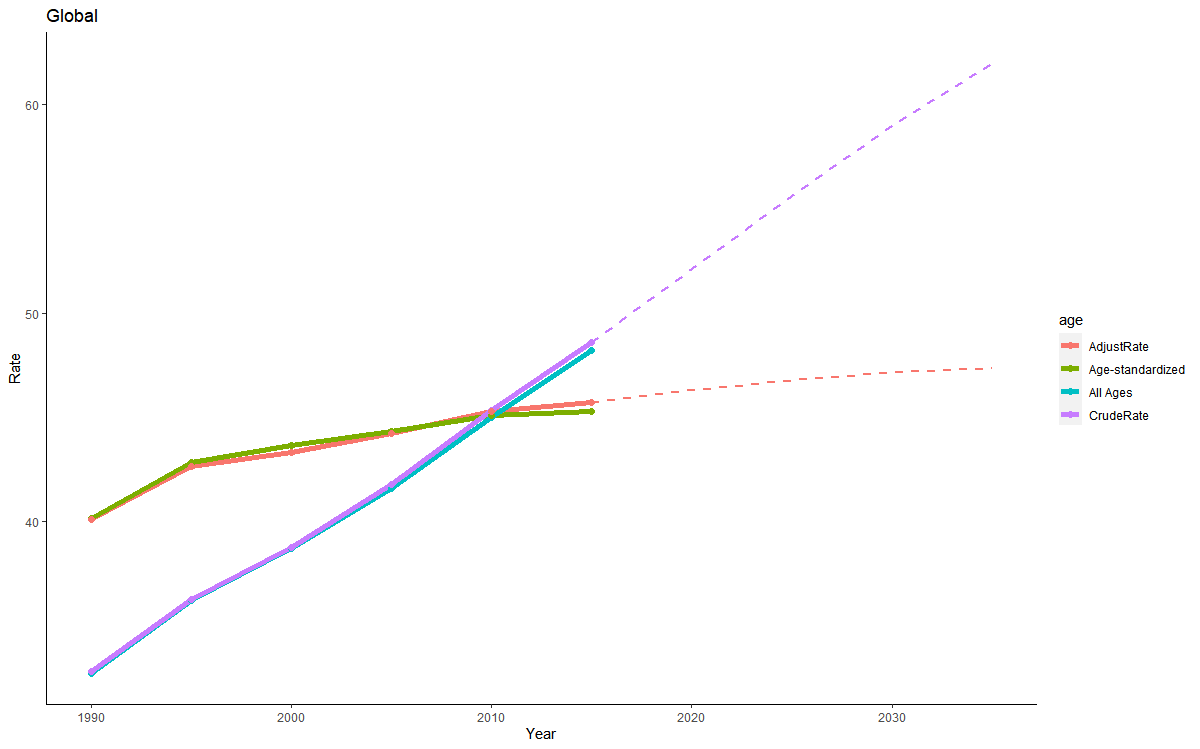

Supplement: Supplementary file 1 [file DataSheet_1.docx]
